# Guideline for veterinary practitioners on canine ehrlichiosis and anaplasmosis in Europe

**DOI:** 10.1186/s13071-015-0649-0

**Published:** 2015-02-04

**Authors:** Ángel Sainz, Xavier Roura, Guadalupe Miró, Agustín Estrada-Peña, Barbara Kohn, Shimon Harrus, Laia Solano-Gallego

**Affiliations:** Department of Animal Medicine and Surgery, Veterinary Clinic Hospital, Faculty of Veterinary Medicine, Universidad Complutense de Madrid, Madrid, Spain; Hospital Clinic Veterinari, Universitat Autònoma de Barcelona, Barcelona, Spain; Department of Animal Health, Veterinary Clinic Hospital, Faculty of Veterinary Medicine, Universidad Complutense de Madrid, Madrid, Spain; Department of Animal Pathology, Faculty of Veterinary Medicine, University of Zaragoza, Zaragoza, Spain; Small Animal Clinic, Faculty of Veterinary Medicine, Freie Universität Berlin, Berlin, Germany; Koret School of Veterinary Medicine, The Hebrew University of Jerusalem, Rehovot, Israel; Departament de Medicina i Cirurgia Animal, Facultat de Veterinaria, Universitat Autònoma de Barcelona, Bellaterra, Spain

**Keywords:** Canine ehrlichiosis, canine anaplasmosis, *Ehrlichia canis*, *Anaplasma phagocytophilum*, *Anaplasma platys*, consensus

## Abstract

Canine ehrlichiosis and anaplasmosis are important tick-borne diseases with a worldwide distribution. Information has been continuously collected on these infections in Europe, and publications have increased in recent years. Prevalence rates are high for *Ehrlichia* and *Anaplasma* spp. infections in dogs from different European countries. The goal of this article was to provide a practical guideline for veterinary practitioners on the diagnosis, treatment, and prevention of ehrlichiosis and anaplasmosis in dogs from Europe. This guideline is intended to answer the most common questions on these diseases from a practical point of view.

## Introduction

Canine ehrlichiosis and anaplasmosis are important tick-borne diseases with a worldwide distribution.

*Ehrlichia canis* was first identified in 1935 in Algeria; dogs infested with ticks showed fever and anemia [1]. Later, during the Vietnam War, many military working dogs brought to Vietnam by the US army exhibited a severe disease called Tropical Canine Pancytopenia [2]. Later, it was renamed canine monocytic ehrlichiosis (CME).

In 1978, an ehrlichial infection that affected platelets was first identified in the US. It was caused by *Anaplasma platys* (initially identified as *Ehrlichia platys*), and it caused a clinical syndrome known as canine infectious cyclic thrombocytopenia [3]. In 1982, a natural infection of granulocytic anaplasmosis, caused by *Ehrlichia phagocytophila,* was first identified in dogs in California. Currently*,* this species is called *Anaplasma phagocytophilum* [4].

Over the 1980’s and 90’s, several species closely related to *E. canis* were found in infected dogs. Improvements in molecular techniques indicated that they were phylogenetically distant from *E. canis*, and therefore, they were reclassified under the genera *Anaplasma* or *Neorickettsia* [5].

In 2002, a large amount of information on ehrlichial infections was gathered by the *American College of Veterinary Internal Medicine* (ACVIM) *Infectious Disease Study Group*. That group published a *Consensus Statement* on diseases caused by *Ehrlichia* spp. in small animals [6]. Over the years, and probably as a result of globalization, urbanization, global warming, increased trade, and travel, these diseases have spread to new niches. Recently, diseases caused by *Ehrlichia* spp have been reported in many countries that had not previously detected them. Information has been continuously collected on these infections, and publications have increased in recent years. This document was intended to answer common questions of interest on this group of infections, with a special focus on the situation in Europe.

## Review

### What species of *Ehrlichia* and/or *Anaplasma* can infect dogs in Europe?

To date, three species have been clearly identified in dogs in Europe; *E. canis*, *A. phagocytophilum*, and *A. platys*.

#### Ehrlichia canis

*Ehrlichia canis* is the only *Ehrlichia* species that has been isolated in dogs from Europe [7,8]. To our knowledge, other *Ehrlichia* species (*E. chaffeensis*, *E. ewingii*, *E. muris*, and *E. ruminantium*) have not been detected in dogs from Europe. However, *E. muris* has been described in *Ixodes* ticks from Russia and Slovakia [9,10]. All European countries bordering the Mediterranean Sea are endemic for *E. canis*. Some surveys have indicated that the infectious agent is spreading to countries north of the Mediterranean (i.e., Switzerland, Germany) [11-13]. Table 1 shows the prevalence of *E. canis* infections in dogs from Europe.

**Table 1:** **Prevalence of** ***E. canis*** **infections in dogs of different European countries**

**Country**	**Number of dogs (n)**	**Prevalence (%)**	**Test methods**	**Reference**
Albania	30	50.0/17.0	IFAT/Blood smear	[14]
Finland	340	0.3	4DX ELISA	[15]
France	919	0.3	4DX ELISA	[16]
Germany	106^a^	0.9	IFAT	[17]
	997^a^	3.1/0	IFAT/PCR	[18]
	4681^a^	10.1	IFAT	[19]
Hungary	1305	0.2	4DX ELISA	[20]
Italy	249^b^	46.0	IFAT	[21]
	135	44.0/6.0	IFAT/PCR	[22]
	51	0	PCR	[23]
	131^b^	22.9	IFAT	[24]
	601	6.4	PCR	[25]
	1000	46.7	IFAT	[26]
	423	20.0	IFAT	[27]
Portugal	557	4.1	4Dx ELISA	[28]
	55	22.0	PCR	[29]
	104	50.0	IFAT	[30]
Romania	1146	2.1	4DX ELISA	[31]
Russia	442^c^ 82^d^	0 2.4	4DX ELISA	[32]
Spain	1100	5.0	4DX ELISA	[33]
	131	5.3	4DX ELISA	[34]
	153	4.0^e^	PCR	[35]
	479	3.1	IFAT	[36]
	466	16.7 4.5	IFAT 3DX ELISA	[37]
	200	6.5	IFAT	[38]
	308	19.2	IFAT	[39]
Switzerland	996^a^	2.2	IFAT	[13]
Turkey	284	20.7	IFAT	[40]

^a^Travelled/imported dogs.

^b^Kenelled dogs.

^c^Dogs with external antiparasitary treatment.

^d^Dogs without external antiparasitary treatment.

^e^Using primers for *Ehrlichia/Anaplasma.*

#### Anaplasma phagocytophilum

Infections of *A. phagocytophilum* have been described mainly in northern and central Europe. Epidemiological studies performed in Europe (Table 2) that evaluated the seroprevalence (rarely a DNA-based analysis) showed that 3 to 57% of dogs carried *A. phagocytophilum*. However, the different studies investigated different dog populations (i.e., healthy and sick), and they used different tests (i.e., immunofluorescent antibody test [IFAT] or enzyme-linked immunosorbent assay [ELISA]). In addition, serological cross-reactivity with other *Anaplasma* spp. (e.g., *A. platys*) can potentially cause an overestimation of the true seroprevalence.

**Table 2:** **Prevalence of infections with** ***A. phagocytophilum*** **in dogs of different European countries**

**Country**	**Number of dogs (n)**	**Prevalence (%)**	**Test methods**	**Reference**
Austria	1470	56.5	IFAT	[41]
Albania	30	40.0/0	IFAT/Blood smear	[14]
Finland	340	5.3	4Dx ELISA^a^	[15]
France	919	2.7	4Dx ELISA^a^	[16]
Germany	1124	50.1	IFAT	[42]
	111	43.2 / 6.3	IFAT/PCR	[43]
	245	19.2	IFAT	[44]
	5881	21.5	4Dx ELISA^a^	[45]
	522	43.0 / 5.7	IFAT/PCR	[46]
	448	19.4	4Dx ELISA^a^	[47]
Hungary	1305	7.9	4Dx ELISA^a^	[20]
Italy	344	0	PCR	[48]
	460	0	PCR	[25]
	5634	33.0	IFAT	[49]
	1232	8.8	IFAT	[50]
	249	38.0	IFAT	[21]
	215	15.0 / 0.02	IFAT/PCR	[51]
Latvia	470	11.4	4Dx ELISA^a^	[52]
Poland	192	1.0	PCR	[53]
Portugal	55	55.0/0	IFAT/PCR	[54]
Romania	1146	5.5	4DX ELISA^a^	[31]
Russia	442^b^ 82^c^	1.1 34.1	4Dx ELISA^a^	[32]
Sweden	611	17.7	IFAT	[55]
	248	20.7	IFAT	[56]
Switzerland	996	7.5	IFAT	[13]
Spain	649	15.6	IFAT	[36]
	466	11.5	IFAT	[37]
	1100	3.1	4Dx ELISA^a^	[33]
United Kingdom	120	0.8	PCR	[57]

^a^This test cannot differentiate between *A. phagocytophilum* and *A. platys.*

^b^Dogs with external antiparasitary treatment.

^c^Dogs without external antiparasitary treatment.

#### Anaplasma platys

*Anaplasma platys* has been diagnosed in dogs, particularly from countries in the Mediterranean basin, including Italy [48], Spain [58], Portugal [59], France [60], Turkey [61], Greece [62], Croatia [63], and Romania [64]. Information is limited regarding the prevalence *of A. platys* infections in dogs from Europe, based on a molecular analysis (Table 3).

**Table 3:** **Prevalence of infections with** ***A. platys*** **in dogs in Europe**

**Country**	**Number of dogs (n)**	**Prevalence (%)**	**Test methods**	**Reference**
Italy	34^a^	52.9	PCR	[65]
	34^a^	70.5	PCR	[66]
	109^a^	27.5	PCR	[67]
	344	4	PCR	[48]
	100^a^	23	RLB-PCR	[68]
Portugal	55^b^	9	IFAT/PCR	[54]

^a^Kenelled dogs.

^b^Dogs with suspected tick-borne diseases.

### What are the vectors and geographic distributions of vectors for these infections in dogs in Europe?

#### Ehrlichia canis

The main, and probably the only, vector for *E. canis* in Europe is the tick *Rhipicephalus sanguineus* sensu lato, known as the brown dog-tick. This tick was shown experimentally to be a competent vector for *E. canis* [69,70]. A recent study has shown that transmission of *E. canis* by *R. sanguineus* ticks starts within 3 h after tick attachment to the dog [71]. The tick vector is currently considered a complex of species [72]. The details of this vector’s morphology and molecular biology have been published, and it has been identified in wide areas of the world. However, it is difficult to ascertain which “species” comprise the actual vector, because the description of the original specimen type has been lost [72].

**Figure 1:**
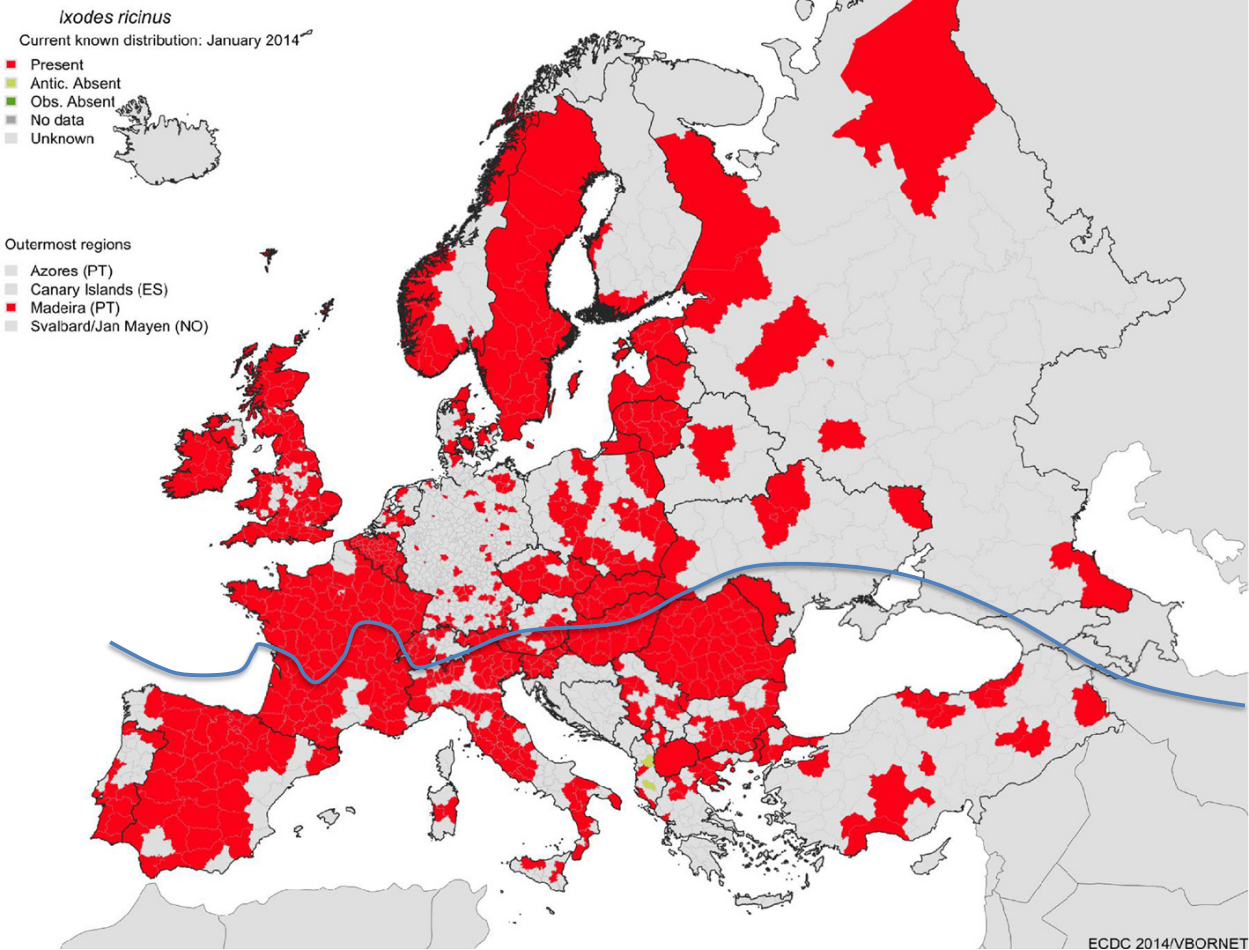
**Distribution of** ***Ixodes ricinus*** **and** ***Rhipicephalus sanguineus*** **in Europe.** The reported distribution of *I. ricinus* was officially recorded by the European Center for Disease Control (ECDC) in January, 2014 (red areas). The map includes only records with reliable, accurate data that can be ascribed to one of the NUTS (statistical regions of Europe). Many reports in the literature only mention the presence of one species in a country. Some of the areas in the Mediterranean region might be based on identified collections that do not have permanent populations (i.e., casual introduction without further survival) or based on small areas of suitability. The line shows the northern range of the distribution of ticks that belong to the *R. sanguineus* complex. Due to the confusion surrounding the morphology and systematics of this complex group, many records cannot be confidently tracked as “true” *R. sanguineus*. This northern limit is also an educated guess for the current distribution, because persistent populations may live in northern latitudes, protected in shelters, burrows, or private gardens, where adequate conditions for survival may exist.

*Rhipicephalus sanguineus* is abundant in the Mediterranean basin. When introduced to colder areas, due to movements of the host (i.e., dogs travelling to and returning from the Mediterranean region), it can survive in kennels and other protected areas provided by man-made constructions (Figure 1). The latitude at which the tick can be established is currently unknown [72,73]. Protected environments, such as a house, a kennel, or the den of a wild carnivore, provide an ideal environment for the reproduction and survival of tick populations. It has been reported that even a single tick female was sufficient to engorge and oviposit on a dog inside an apartment, which then could give rise to the development of subadults [74]. *Rhipicephalus sanguineus* populations can reach very high numbers in sheltered environments, because the blood supply necessary for their development is guaranteed by the presence of hosts in close proximity. In dogs without appropriate protection, parasitic loads can reach hundreds of ticks per animal, with ticks in all developmental stages.

The tick requires a minimum temperature of about 6°C for adequate survival, and when temperatures drop below this value, it may hibernate during winter, sheltered in the cracks of kennels and buildings. Ticks also require a certain level of humidity, which is provided by the high environmental moisture in the kennels (cleaned with water) or in gardens that receive artificial watering. Ticks are common and can reach significant population densities on riverbanks of the Mediterranean region, which are temperate and hold moisture. The tick is mainly active from spring to early autumn, when the highest infestations occur. The regional weather or the local conditions of some buildings may result in a different phenology or in different seasonal dynamics for the tick [72]. The impact of climate trends on the tick is unknown, although preliminary results have shown that their area of distribution is increasing, due to the increase in urbanized areas surrounding cities, and due to the warmer environment observed in autumn and winter at progressively higher latitudes.

#### Anaplasma phagocytophilum

*Ixodes ricinus* is the only known vector for *A. phagocytophilum* in Europe. This tick is widely distributed in most European regions, including western Norway and in Sweden; it has been collected in mixed forests, open pastures, and other areas with high humidity (Figure 1). The driving feature of the northern distribution is the temperature required for tick development. In the northern areas of Sweden, the tick was found at an approximate latitude of 68°N, but some tick populations have spread even farther north [75]. *Ixodes ricinus* is present in Ireland, the United Kingdom, and in southern Finland. It is also present in almost every territory in central Europe, and the eastern limit seems to be along the Baltic countries and Ukraine. At its present limit to the east, it coexists with the closely-related, species *Ixodes persulcatus*, with which there is a certain degree of sympatry. In the south, the tick is present in parts of the Mediterranean region, commonly in mixed or deciduous forests [76,77]. It has been detected in Portugal, Spain (only in northern areas of the country and isolated points in southern hills), and Italy. It is also common in the northern belt of humid forests in Turkey [76,77]. It is hypothesized that the driving feature of its southern distribution is the atmospheric water vapour, which must be relatively high (around 80%) to support permanent populations.

In the eastern regions of Europe, *I. ricinus* is replaced with *I. persulcatus*, a closely related species. The habitats of *I. persulcatus* extend to the east and into Japan [78]. This well-known parasite inhabits a wide range of domestic animals, and it acts as a vector for different pathogens. It is probably best known as the main vector for *Borrelia* spp. and for tick-borne encephalitis virus in the territories it colonizes [79]. *I. persulcatus* was found to be infected by *Ehrlichia* spp. in the Baltic region [80] and by *A. phagocytophilum* in Ukraine [81]. Members of the Anaplasmataceae family (without further description) have also been found in *I. persulcatus* collected in Moldova [82].

#### Anaplasma platys

The probable vector of *A. platys* in Europe is *R. sanguineus*. Experimental transmission has not been achieved [83]. Nevertheless, molecular studies have detected *A. platys* DNA in *R. sanguineus* ticks, which suggested that these ticks act as the vector for *A. platys* [84].

### Are there other routes of transmission for these infections in dogs?

Outside the European continent (on which this guideline is focused), other species of ticks have been shown to be responsible for the transmission of *E. canis* [85]. However, to date, *R. sanguineus* appears to be the main vector for this pathogen.

In general, another route of transmission is through blood transfusion. Due to the potential transmission of these pathogens via infected blood, screening canine blood products for bacterial DNA with a polymerase chain reaction (PCR) assay is recommended in highly endemic areas to ensure the safety of blood products [86]. This route of transmission has been described for *E. canis* [69], *A. phagocytophilum* [87-89], and *A. platys* [90].

### Are *Ehrlichia* and *Anaplasma* species present in dogs in Europe able to affect other hosts?

#### Ehrlichia canis

Other wild canids (foxes, wolves, jackals) can become infected with *E. canis* [91-93]. However, experimental infection has only been reproduced in dogs. In addition, *E. canis*-like DNA was claimed to be detected in cat samples with PCR [94]. Moreover, several studies reported the presence of *E. canis* DNA in cats and wild felids from Brazil and Portugal [95-97]. However, to the best of our knowledge, *E. canis* has not been isolated from cats or other felids to date.

#### Anaplasma phagocytophilum

*Anaplasma phagocytophilum* can infect humans and several animals other than dogs, including cats, sheep, goats, cows, equines, rodents, roe deer, deer, other wild mammals, and birds [98-100].

#### Anaplasma platys

*Anaplasma platys* DNA was also recently found in cats in non-European continents [101,102].

### What is the public health importance of *E. canis*, *A. phagocytophilum*, and *A. platys*?

#### Ehrlichia canis

An organism closely related to *E. canis* has been described in humans in Venezuela [103]. To date, it is not considered an agent with important zoonotic potential.

#### Anaplasma phagocytophilum

*Anaplasma phagocytophilum* has important zoonotic potential, because humans can acquire the infection through a bite from a tick infected with the pathogen. *Anaplasma phagocytophilum* causes human granulocytic anaplasmosis, a febrile illness that closely resembles the disease in dogs. After infection (1-2 weeks), a syndrome frequently occurs of malaise, low-back pain, fever, and/or gastrointestinal disorders. Other, less frequent symptoms include arthralgia, lymphadenomegaly, conjunctivitis, dysuria, and peripheral edema [104,105].

The first infection by *A. phagocytophilum* in humans was described by Bakken *et al* in 1994 in the USA [106]. After that description, numerous human cases from different European countries were described [107]. Dogs may act as sentinels for human exposure, because infected ticks on their coats may travel to humans. Blood from infected dogs should be handled with caution [107,108].

#### Anaplasma platys

*Anaplasma platys* DNA was recently detected in two family members in the USA [109] and two women from Venezuela [110].

Other *Ehrlichia/Anaplasma* spp. that have not been described in Europe can also infect humans. *Ehrlichia ewingii* has been described as a cause of infection in immune-suppressed patients [111], but there is no evidence of direct or tick-mediated transmission from dogs to human beings. *Ehrlichia ruminantium*, which typically infects wild ruminants, is also able to cause a disease in dogs [112] and humans [112,113]. *Ehrlichia chaffeensis* is the etiologic agent of human monocytic ehrlichiosis [99]. *Amblyomma americanum* acts as a vector for *E. chaffeensis* in the USA. Its hosts include white-tailed deer, goats, domestic dogs, red foxes, and birds. This *Ehrlichia* sp. has also been described in dogs [114,115], and DNA of *E. chaffeensis* has been found in ticks collected from dogs [116]. Finally, it has been shown that *E. muris* can infect both humans and dogs [117,118].

### Is there a breed, age, or sex predisposition for canine ehrlichiosis or anaplasmosis?

#### Ehrlichia canis

All breeds are prone to CME. However, German Shepherd dogs and Siberian Huskies are predisposed to develop more severe clinical signs of ehrlichiosis; therefore, these breeds have a worse prognosis [119,120]. This fact was corroborated in experimental studies, which showed that the cell-mediated immune response to a challenge with *E. canis* was reduced in German Shepherd dogs compared to Beagle dogs [119].

Canine ehrlichiosis may appear at any age. No sex predisposition has been detected in the development of CME. Some studies have found higher seropositivity in males, but this may be explained by a higher exposure to vectors than females, due to behavioral characteristics [121]. Similarly, in epidemiological studies, higher seropositivity rates were found in older dogs [121-123]; this observation may be attributed to a higher probability of exposure to *E. canis* as the dog ages, rather than an increase in susceptibility with age.

#### Anaplasma phagocytophilum

No breed predisposition has been confirmed for *A. phagocytophilum* infections. In a few studies, Retriever breeds were overrepresented; however, this might reflect the popularity of taking these dogs along for outdoor activities [124]. No sex or age predisposition has been documented for clinical cases. Older dogs were more often seropositive compared to younger dogs, probably due to an increased opportunity for exposure over time [46,55].

#### Anaplasma platys

No breed, age, or sex predisposition has been described for *A. platys* infections.

### What stages of disease can be identified during infections with *Ehrlichia* and/or *Anaplasma* spp. in dogs?

#### Ehrlichia canis

Following an incubation period of 1 to 3 weeks, three typical phases of the disease may develop sequentially: acute, subclinical, and chronic [2,125]. The acute phase can last 2 to 4 weeks; then, clinical signs may vary or disappear spontaneously, even without treatment. However, some dogs that show clinical improvement may remain persistent subclinical carriers for months and years [126]. The subclinical phase follows the acute stage. Dogs in this stage present no clinical signs; therefore, they may not seem to require veterinary attention. However, when screened for hematological abnormalities, these dogs may have subnormal platelet concentrations [127]. Some, but not all infected dogs may advance to a chronic phase. In the chronic phase, the clinical signs are more severe. Unfortunately, the acute and chronic phases are not always easy to distinguish in clinical practice, because many of the clinical signs are similar. A complete blood count and bone marrow aspiration may assist in diagnosing the chronic severe form of the disease. Dogs in the chronic phase exhibit bone marrow hypoplasia and severe pancytopenia [128]. No studies have fully elucidated the different responses of dogs to infection or the factors that induce some dogs to develop the chronic severe form of the disease.

#### Anaplasma phagocytophilum

The incubation period for an *A. phagocytophilum* infection lasts 1 to 2 weeks. Thereafter, the dog may develop a self-limiting febrile illness [87]. The main difference between *A. phagocytophilum* and *E. canis* is that most *A. phagocytophilum* cases present in the acute stage of the disease. Indeed, most dogs naturally infected with *A. phagocytophilum* probably remain healthy, as indicated by the high number of healthy seropositive dogs relative to dogs with the clinical disease [46]. The extent to which *A. phagocytophilum* can persist in tissues and contribute to subclinical/chronic disease manifestations in dogs has been a controversial issue [129].

Three dogs experimentally inoculated with *A. phagocytophilum* were monitored for 4 to 6 months to determine whether the organisms persisted. Rickettsial DNA was detected intermittently in two dogs, when PCR was performed on blood samples throughout the study [129].

#### Anaplasma platys

The incubation period for an *A. platys* infection is similar to that of an *A. phagocytophilum* infection (1 to 2 weeks). After that, alternate periods of thrombocytopenia and fever are observed, which appear and disappear cyclically every 1-2 weeks [130].

After an experimental inoculation of dogs with *A. platys*, parasitized platelets were detected in the peripheral blood after 8 to 15 days. The dogs developed severe thrombocytopenia within 7 days post inoculation [90]. After a reduction in the number of circulating bacteria, the platelet concentration increased within 3-4 days. These episodes of bacteremia and thrombocytopenia occurred at 1-2 week intervals. Chronic infection is associated with low-level bacteremia and mild thrombocytopenia, which may reflect a process where the host attempts to adapt to the *A. platys* infection [131].

### What clinical signs are associated to *Ehrlichia* spp. and *Anaplasma* spp*.* infections in dogs?

#### Ehrlichia canis

Clinical signs of an *E. canis* infection can be variable, depending on the strain, the immune response of the dog, and the presence of concomitant infections with other tick- or flea-borne pathogens. It must be kept in mind that some dogs may not show clinical or laboratory signs associated with an *Ehrlichia* infection, and others may show severe signs. In general, *E. canis* seems to cause more severe clinical signs than anaplasmosis [3,35,87,120,124,132-142].

Clinical signs of ehrlichiosis can vary, and it may include nonspecific signs, like fever, weakness, lethargy, anorexia, lymphadenomegaly, splenomegaly, hepatomegaly, or weight loss. Other signs have also been described, including vomiting, diarrhea, pain, exercise intolerance, edema (in hind legs, tail, or scrotum), cough and/or dyspnea (associated with pneumonia), serous or mucopurulent oculonasal discharge, abortion or neonatal death, and skin ulcers. Although some older studies have included lameness, due to polyarthritis, as a sign of CME [143,144], no evidence-based studies have supported this sign. Therefore, in cases of lameness and/or polyarthritis in dogs infected with *E. canis*, it is advisable to rule out coinfections with other pathogens, such as *A. phagocytophilum* or *B. burgdorferi*.

Common clinical signs of ehrlichiosis include pale mucous membranes, due to anemia, epistaxis, petechiae, ecchymoses, prolonged bleeding during estrus, hematuria or melena associated with thrombocytopenia, thrombocytopathy, or vasculitis. Ocular signs are also common in CME. The most common are anterior uveitis, corneal opacity, hyphema, retinal vessel tortuosity, chorioretinal lesions, subretinal hemorrhage, retinal detachment, or blindness. Neurological signs are less commonly described (typically secondary to meningitis).

#### Anaplasma phagocytophilum

The most common clinical signs of *A. phagocytophilum* are non-specific; they include lethargy, inappetence/anorexia, and fever. Other findings are pale mucous membranes, a tense abdomen, and gastrointestinal signs (vomiting/diarrhea). Lameness may result from secondary immune-mediated (neutrophilic) polyarthritis. Mildly enlarged lymph nodes, tachypnea, and surface bleeding (petechiae, melena, epistaxis) may occur. Rare signs include collapse, mild cough, scleral injection, uveitis, limb edema, and polydipsia/polyuria [145-151]. It is controversial whether central nervous system signs are associated with this infection [152-154]. In a few cases, an association was suggested between steroid-responsive meningitis/arteritis and *A. phagocytophilum* infection [155]. *A. phagocytophilum* infection may trigger some immunopathies, such as immune-mediated thrombocytopenia/anemia. Splenomegaly diagnosed with radiography and ultrasonography is a very common finding [147].

#### Anaplasma platys

Clinical signs of *A. platys* infection have been described both experimentally and in naturally infected dogs in Greece, Spain, Portugal, and Israel. These signs included fever, lethargy, anorexia, weight loss, pale mucous membranes, petechiae, nasal discharge, and lymphadenomegaly. Furthermore, single case studies have described bilateral uveitis and epistaxis. However, not all studies used PCR to exclude co-infections with other vector-transmitted diseases that produce the same clinical signs [54,62,63,66,146,156-158].

### What abnormal laboratory findings can be found in dogs with ehrlichiosis and/or anaplasmosis?

Abnormal laboratory findings in canine ehrlichiosis or anaplasmosis are variable and nonspecific. However, the most common finding in ehrlichiosis and anaplasmosis is thrombocytopenia; this finding should alert clinicians. Other common abnormal laboratory findings are shown in Table 4 [54,147,148,159].

**Table 4:** **Laboratory abnormal findings in ehrlichiosis caused by** ***Ehrlichia canis*** **and anaplasmosis caused by** ***Anaplasma platys*** **and** ***Anaplasma phagocytophilum***

**Clinical disease**	**Species**	**Laboratory abnormal findings**
Canine monocytyc ehrlichiosis	*E. canis*	• Mild to moderate normocytic normochromic non regenerative anemia
		• Neutropenia
		• Neutrophilia
		• Lymphopenia
		• Monocytosis
		• Granular lymphocytosis (uncommon)
		• Thrombocytopenia
		• Thrombocytopathy
		• Pancytopenia: medullary hypoplasia or aplasia (chronic forms, 15-20% of clinical cases)
		• Hyperproteinemia
		• Hyperglobulinemia
		• Hypergammaglobulinemia, usually polyclonal
		• Hypoalbuminemia
		• Proteinuria
		• Renal azotemia
		• Mild increase in hepatic enzymes (ALT, ALP)
		• Mononuclear or neutrophilic pleocytosis
Canine granulocytic anaplasmosis	*A. phagocytophilum*	• Non regenerative mild to moderate normocytic normochromic anemia
		• Regenerative anemia (rare)
		• Lymphopenia
		• Neutropenia, neutrophilia (sometimes left shift) or normal neutrophil concentration
		• Thrombocytopenia
		• Hyperglobulinemia
		• Hypoalbuminemia
		• Increased ALP
		• Mild hyperbilirubinemia
		• Neutrophilic inflammation in synovial fluid
		• Sometimes direct Coombs’ test/platelet-bound antibody test positive
Canine thrombocytotrophic anaplasmosis	*A. platys*	• Thrombocytopenia
		• Non regenerative mild to moderate normocytic normochromic anemia
		• Hyperglobulinemia
		• Hypoalbuminemia

### Is microscopic evaluation of a blood smear or another tissue sample useful for the diagnosis of ehrlichiosis and anaplasmosis?

The diagnostic usefulness of evaluating a blood smear depends on the pathogen species that has infected the dog.

#### Ehrlichia canis

**Figure 2:**
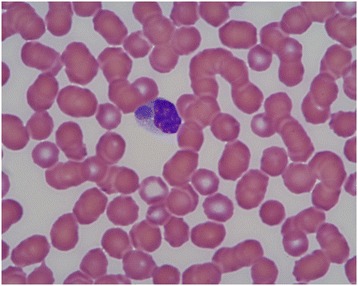
**Microscopic image of a morula of** ***Ehrlichia canis*** **in the cytoplasm of a monocyte (x100).**

It is rare to detect an *E. canis* morula (an aggregate of *E. canis* organisms; Figure 2) in a blood smear. It occurs in about 4-6% of clinical cases [160]. The highest likelihood of detecting morulae can be achieved by performing a buffy coat smear [160]. Because the direct detection method has low sensitivity, further diagnostic tests must be conducted, such as serology or molecular techniques (PCR). The highest rates of detecting morulae (50%) seem to be achieved when lymph node aspirates are examined by expert cytologists that evaluate a large number of oil immersion fields [160,161].

#### Anaplasma phagocytophilum

**Figure 3:**
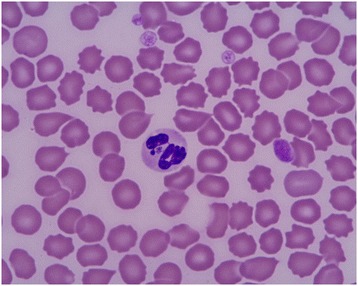
**Microscopic image of a morula of** ***Anaplasma phagocytophilum*** **in the cytoplasm of a neutrophil (x100).**

The direct blood smear detection method seems to have higher sensitivity for detecting *A. phagocytophilum* than for detecting *E. canis* in dogs. Morulae can be observed in neutrophils in up to 60% of clinical cases [147] (Figure 3). In experimentally infected dogs, morulae appear as early as 4 days after inoculation and persist for 4-8 days [129]. However, the morulae cannot be distinguished from those of other *Ehrlichia* spp. that infect neutrophils (e.g., *Ehrlichia ewingii*); therefore, the finding should be confirmed by PCR in areas where both organisms are likely to occur.

#### Anaplasma platys

**Figure 4:**
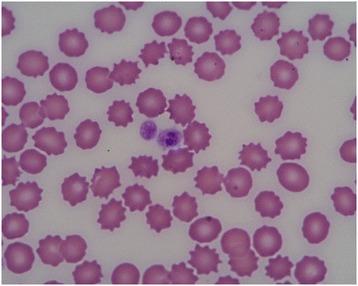
**Microscopic image of a morula of** ***Anaplasma platys*** **in a platelet (x100).**

The method of detecting morulae in platelets during an *A. platys* infection also appears to have a low sensitivity (Figure 4) [57], but some studies suggest that this method may have higher sensitivity during the early phase of the infection [162]. It was recently reported that *A. platys* were found in megakaryocytes [163], but the sensitivity of bone marrow cytology remains to be evaluated.

### What serological techniques can be used in the diagnosis of ehrlichiosis and/or anaplasmosis?

Ehrlichiosis and/or anaplasmosis can be diagnosed with the IFAT or ELISA [142,164,165]. These tests require specific equipment and trained technicians. One of the advantages of these tests is that they allow determination of antibody levels and their changes over time. Therefore, it is important to conduct quantitative serologic tests and to determine either the final antibody titer with the IFAT or the optical density with the ELISA for a quantitative evaluation (negative, low, or high signals). Quantitative laboratory techniques are more sensitive and specific than rapid tests [164]. Some commercial in-house dot-ELISA kits are qualitative, and show only a positive or negative result, without providing the dog’s antibodies levels. Other dot-ELISA kits are semi-quantitative and provide some information on antibody levels. However, an accurate, reliable serological diagnosis is limited by the lack of standardization among different diagnostic laboratories and tests.

### How should we interpret a positive serologic result for *Ehrlichia* and/or *Anaplasma*?

A positive serologic result indicates a past or current infection, but it does not always denote an ongoing disease condition. A single positive titer result may only reflect a past infection that may be resolved, because antibody titers may persist for several months or years [46,90,126]. Moreover, seroprevalence is high in endemic areas. In dogs naturally infected with *E. canis*, some authors have found that animals with high IFAT titers were more likely to show PCR positivity than negativity; this finding suggested that there may be a relationship between antibody titer and active infection [166]. Nevertheless, this information must be confirmed. We suggest that suspected cases should be evaluated based on the performance of two or more serological tests conducted in 2-4 weeks intervals. This approach can provide information on antibody kinetics (an increase, no change, or a decrease), which may point to the current status of infection. It has been suggested that a four-fold increase in IgG antibodies over time can be taken as evidence of an ongoing infection [165]. Recent research has suggested that the combination of serology and PCR was preferable for the diagnosis of *Ehrlichia* and *Anaplasma* spp. infections [167]. Presently, we recommend performing molecular tests to support any positive serological case, because the presence of bacterial DNA is a sign of active infection.

### Could a seronegative dog be infected with *Ehrlichia* and/or *Anaplasma* spp.?

A dog can be seronegative, despite harboring an infection, with some species of *Ehrlichia* or *Anaplasma*. This is particularly common during the incubation period and in the early stages of the acute disease, when bacterial loads are low, for example:

- In the case of *E. canis*, antibody production typically does not occur before 12 -14 days post-infection [126,168].
- After experimentally infecting dogs with *A. phagocytophilum*, IgG antibodies may first be detectable 8 days after the initial exposure and 2 to 5 days after the appearance of morulae [129].
- After experimentally infecting dogs with *A. platys*, antibodies were first detected on day 16 [90].

Consequently, seroconversion can be used as a serologic technique to corroborate signs that suggest an acute infection with *Ehrlichia* or *Anaplasma*. Thus, an initial quantitative serology is conducted to detect acute antibody levels, when the dog shows clinical signs and/or abnormal laboratory findings; then, another quantitative serology is conducted to detect convalescent antibody levels after 2-4 weeks. Positive antibody levels in the convalescent stage confirm the infection for some species of *Ehrlichia* and/or *Anaplasma* at disease onset. It should be noted that the administration of doxycycline will take effect over days and weeks; therefore, it will have no impact on the production of antibodies or the seroconversion [90,168-171].

### Do serological cross reactions exist among *E. canis*, *A. phagocytophilum*, and *A. platys*?

It is generally accepted that no important cross reaction exists between *Ehrlichia* and *Anaplasma*. However, a potential cross reaction has been described between *E. canis* and *A. phagocytophilum*, particularly when one of the pathogens is present at very high titers or when the follow up is prolonged [37,165]. Apparently, there is no serological cross reaction between *E. canis* and *A. platys*; however, only a few, limited serologic studies have investigated this possibility [172].

When antibodies cross-react with more than one antigen/pathogen, the antibody titers that are highest indicate the most probable infecting pathogen in the dog [37]. However, a cross reaction was described between *A. phagocytophilum* and *A. platys* [37,173]. Moreover, *A. phagocytophilum* might cross react with other non-ehrlichial species (e.g., *Coxiella burnettii*) [54,145].

As serological cross reactions may occur between different *Ehrlichia* and *Anaplasma* species, the serology should be complemented with molecular techniques, such as PCR and DNA sequencing.

### Why is a polymerase chain reaction assay useful in the diagnosis of these diseases?

PCR is very useful in diagnosing these infectious diseases for several reasons. First, PCR detection is more sensitive than a direct microscopic examination. Second, the detection of DNA for a specific pathogen in a clinical setting should be considered evidence of an active infection. Third, real-time PCR permits quantification of bacterial loads. Finally, PCR allows one to investigate specific gene fragments after amplification. Sequencing the gene fragment/s amplified by PCR may reveal the identification of the specific *Ehrlichia*/*Anaplasma* species that infected the dog.

Real-time PCR assays that were developed for detecting infections of *Ehrlichia* or *Anaplasma* in peripheral blood have provided high sensitivity, similar to the sensitivity previously obtained with splenic samples [169,174]. Several third-party laboratories provide various PCR assays for detecting *Ehrlichia* and/or *Anaplasma* spp. However, the results depend on the experience and quality of the laboratory. The use of non-specific primers and low annealing temperatures in some PCR assays can result in non-specific amplification; thus, the evaluation may provide misleading, false-positive results.

Finally, it must be kept in mind that false-negative results can also occur due to the absence of pathogens in the sample. For example, bacteremia may be intermittent in some dogs or in specific diseases (e.g., *A. platys* infection); it may be present below the minimal detection level of the assay [174,175]; or it may be absent, due to previous administration of antibiotics, such as doxycycline. Therefore, a negative PCR result should be interpreted as “no detection of the tested pathogen-DNA in the sample”, rather than “the sample is free of the tested pathogen”. Thus, to improve the usefulness of PCR and obtain as much information as possible from the results, it is important to submit samples to reliable laboratories and to assess the results in combination with an evaluation of antibody titers, clinical signs, and abnormal laboratory findings.

### What samples should be chosen to perform a molecular diagnosis of *Ehrlichia* and/or *Anaplasma* spp. infections?

The sample of choice for a PCR assay is peripheral blood, buffered with EDTA. Some studies have suggested that splenic aspirates seem to be optimal specimens for the diagnosis of *E. canis* infections based on PCR [176]. When microscopic examination reveals structures suggestive of morulae in the cytoplasm of neutrophils or mononuclear cells in any tissue or fluid, PCR can be used to corroborate the diagnosis.

### Are coinfections common in dogs infected with *Ehrlichia* and/or *Anaplasma* spp.?

Coinfections with *Ehrlichia* and/or *Anaplasma* spp. are common, because some species are transmitted in the same arthropod vector [145,177]. Accordingly, coinfection of *E. canis* with *A. platys* should occur frequently.

Currently, many other vector-borne diseases have been shown to coexist in the same dog, including other tick-borne pathogens (*Babesia* and *Hepatozoon* spp.), Culicidae mosquito-borne pathogens (*Dirofilaria* and other filariae), sand fly-borne pathogens (*Leishmania infantum*) [33,162], and even intestinal parasites [178]. In the Mediterranean basin, the most common coinfecting agents are *L. infantum* and *Babesia* spp*.* Less common are coinfections with *Hepatozoon* spp.

Due to the shared arthropod vectors and/or concurrent exposure to multiple vector-carrying ticks, co-infections may occur with other tick-borne pathogens (e.g., *Borrelia*, *Bartonella*, *Rickettsia*, *Babesia*, and arboviral spp.), which can complicate the clinical picture [179]. Because *Borrelia burgdorferi* and *A. phagocytophilum* are transmitted by the same ixodid tick species, coinfections with these two pathogens are frequently detected. Moreover, the two organisms may enhance one another’s pathogenicity [180]. In northern California, dogs that are seroreactive to *A. phagocytophilum* were 18 times more likely to be seropositive for *Bartonella vinsonii* subs. *berkhoffii* than dogs seronegative for *A. phagocytophilum* [181].

### What is the clinical significance of a coinfection during the course of the disease?

Coinfections tend to worsen some conditions and mask others, which could complicate the diagnosis [131]. Most vector-borne diseases have the special feature of causing similar clinical signs and abnormal laboratory findings in dogs (e.g., epistaxis and uveitis are signs that can be due to ehrlichiosis and/or leishmaniosis). This similarity complicates the diagnosis and increases the list of differential diagnoses. In one study, a coinfection with CME and *Leishmania* sp. delayed clinical improvement compared to dogs with either CME or leishmaniosis alone [182]. In another study, more clinical signs were noticed in dogs with dual infections (*E. canis* and *L. infantum*), which supported the notion that the pathogens may have synergistic pathological effects [183]. Furthermore, the occurrence of *A. platys* together with other infectious agents (e.g., *E. canis*, *Babesia vogeli*, or *Rickettsia conorii)* may potentiate the clinical disease and the laboratory abnormalities [54,66,90].

Therefore, when an infection of one specific species is diagnosed, a comprehensive work-up is necessary to rule out coinfections with other vector-borne pathogens [177].

### What is the treatment of choice for *Ehrlichia* spp. and *Anaplasma* spp. infections?

#### Ehrlichia canis

CME is usually successfully treated with antibiotics that belong to the tetracycline family. The treatment of choice is doxycycline at 5 mg/kg twice daily or 10 mg/kg once daily for 4 weeks. This regime guarantees a complete response in the majority of cases [125,168,176]. Some reports described experimentally infected dogs that remained infected and became subclinical carriers after shorter treatments with doxycycline, even at the recommended doses [125,166,170]. Therefore, the more prolonged treatment of 4 weeks is recommended.

Although clinical improvement is achieved in most cases, it can be difficult to ensure treatment effectiveness regarding the total elimination of *Ehrlichia* spp. in naturally infected dogs. The clinician should not target seronegativity with treatment, but rather, negative PCR results [6,142,166,168].

Doxycycline, unlike other tetracyclines, does not seem to cause enamel discoloration in puppies. Moreover, one of the most common side effects of tetracyclines, vomiting, can be eliminated by splitting the antibiotic dose into two half-doses every 12 h (q12h) or by administering the antibiotic after feeding. When the dog has liver problems, the use of doxycycline should be reconsidered. In any case, liver function tests should be performed before and during treatment; when liver parameters increase, the treatment regime should be stopped [6,142,166,168].

Other drugs have been historically used for treating CME. Chloramphenicol has been used in dogs under 1 year of age, but its use is not recommended when doxycycline is available. Imidocarb dipropionate has also been described as a potential treatment for ehrlichiosis in dogs [171,184,185]. Recent studies performed *in vitro* and in experimentally infected dogs showed that imidocarb dipropionate was not effective against *E. canis* [186,187]. It should only be used in cases of co-infection with *Babesia canis* or *Hepatozoon canis*. Other antibiotics, such as rifampicin or levofloxacin, have been effective in *in vitro* studies [188]. However, recent studies have shown that, in experimental infections, rifampicin contributed to improving laboratory findings, but it was not effective in eliminating the infection [189].

#### A. phagocytophilum

Doxycycline (for 2-3 weeks) at the dosage described above for *E. canis* is apparently effective in treating *A. phagocytophilum* infections. However, several parameters remain to be established, including the most effective or optimal dose; the length of doxycycline treatment; and the value of adding other antibiotics [145,146,174,175,190]. Rifampin and levofloxacin have also shown *in vitro* effects against *A. phagocytophilum* [190]. Chloramphenicol has been mentioned as an alternative for treating puppies [191].

When dogs with circulating *Anaplasma* organisms have moderate to severe clinical disease or do not rapidly respond to doxycycline therapy, they should probably be tested for other tick-borne diseases [146].

#### Anaplasma platys

*Anaplasma platys* infections can be treated with tetracyclines (e.g., doxycycline 5-10 mg/kg q12-24h for 8-10 days). Alternatively, they can be treated with enrofloxacin at 5 mg/kg, q12h for 14-21 days [131,192].

### Are there other supportive therapies that could be used for ehrlichiosis and/or anaplasmosis?

In severe or chronic ehrlichiosis and/or anaplasmosis, other therapeutic agents or treatments may be needed, particularly for *E. canis* infections. For cases with hemorrhage or organic lesions, the dog may require admission to a hospital for a more intensive treatment. In general, according to the seriousness determined in the clinic, dogs may need blood transfusions when the packed cell volume (PCV) is very low; fluid therapy when dehydration or secondary kidney disease is present; or antipyretic and analgesic drugs [138,142,146].

In some cases of chronic ehrlichiosis, when aplasia of all cell lines occurs, other treatments have been used. For example, growth factors, like granulocyte colony stimulating factor or erythropoietin have been used; however, scientific evidence of effectiveness is scarce. One case of chronic ehrlichiosis was treated successfully with a combination of hematopoietic growth factors, low dose vincristine, doxycycline, and glucocorticoids [193].

### Is it necessary to use steroids for treating ehrlichiosis and/or anaplasmosis?

Treatment of ehrlichiosis or anaplasmosis should not be initiated with glucocorticoids combined with antibiotics. Steroids should only be considered when no satisfactory response is noticed, or when immune-mediated complications arise. *Ehrlichia* and *Anaplasma* species may mediate an immune response, typically indicated by hemolytic anemia, thrombocytopenia, uveitis, glomerulonephritis, vasculitis, etc. In those cases, treatment with glucocorticoids (usually prednisone or prednisolone) may be indicated; doses of prednisone should range from 0.5 to 2 mg/kg/day, and the treatment duration should vary according to the type and seriousness of the associated immune-mediated condition [133,147,152,194-196]. However, the use of immunosuppressive glucocorticoids for treatment of secondary immune-mediated diseases must be considered very carefully. Of note, the administration of immunosuppressive doses of glucocorticoids or other drugs to subclinically infected dogs may result in the re-emergence of bacteremia, although the dog may not show clinical signs [6,129,142,174,175].

### What is the expected clinical response following the treatment of ehrlichiosis and/or anaplasmosis?

Doxycycline is an effective drug against ehrlichiosis and/or anaplasmosis in dogs [120,145,146]. When dogs are treated in the acute phase of ehrlichiosis and anaplasmosis, they improve quickly, within 24-48 h, and their prognosis is good when the whole course of therapy is administered [120,147,197].

However, the prognosis of dogs with the chronic severe form of ehrlichiosis is grave [120,197]. Shipov and colleagues (2008) investigated prognostic indicators for mortality and survival in canine ehrlichiosis [198]. They found that pronounced pancytopenia (white blood cells [WBC] < 4 × 10^3^/μL; PCV < 25%; and platelet concentration [PLT] < 50 × 10^3^/μL) was a risk factor for mortality. In that study, they found that severe leucopenia (WBC < 0.93 × 10^3^/μL), severe anemia (PCV < 11.5%), prolonged activated partial thromboplastin time (APTT > 18.25 s), and hypokalemia (K < 3.65 mmol/L) could each predict mortality with a probability of 100%. Conversely, a WBC > 5.18 × 10^3^/μL, PLT > 89.5 × 10^3^/μL, PCV > 33.5%, APTT < 14.5 s, or K > 4.75 mmol/L could each predict survival with a probability of 100%.

When the clinical response in canine ehrlichiosis or anaplasmosis is not rapid, or when clinical signs persist after treatment with doxycycline, the dog must be re-examined for other infectious diseases or diseases with similar clinical signs (neoplasia or immune-mediated diseases) [6,142,146].

### How do abnormal laboratory findings evolve in dogs infected with ehrlichiosis/anaplasmosis after the treatment?

#### Ehrlichia canis

Laboratory abnormalities in canine ehrlichiosis typically resolve in dogs after treatment with acute disease. In general, both the complete blood count and the alterations in serum protein electrophoresis should normalize within 10-15 days after therapy initiation [6,142].

Chronic changes, such as mild or moderate hyperglobulinemia, anemia, or thrombocytopenia may persist as long as the infectious agent has not been completely eliminated, or when some other associated conditions exist [171]. In naturally infected dogs, despite evident clinical improvement, it is difficult to guarantee treatment effectiveness and complete elimination of the involved pathogens [6,142]. When the dog no longer produces specific antibodies to the pathogen, it can be assumed that complete elimination of the *Ehrlichia* species has been achieved [120].

Quantification of acute phase proteins in serum has been assessed for its value in monitoring and evaluating the prognosis of dogs with ehrlichiosis. Although quantification of acute phase proteins may be a useful indicator of the clinical stage, it has no predictive value for the final clinical outcome [199].

Finally, it must be recognized that prior infections with *E. canis* do not confer permanent immunity to dogs. Therefore, dogs can be re-infected with the same pathogen or with other species after re-exposure to infected ticks [6,142].

#### Anaplasma phagocytophilum

Similar to canine ehrlichiosis, after treating anaplasmosis, laboratory alterations in the complete blood cell count and in serum protein electrophoresis should be resolved in most dogs [146,147].

#### Anaplasma platys

Thrombocytopenia in dogs infected with *A. platys* typically disappears around one week after starting therapy [192].

### How do antibody titers evolve following treatment?

#### Ehrlichia canis

Antibody titers may persist for months. Then, in most *E. canis* infections, they decrease gradually over 6-9 months post treatment. Sometimes, antibodies are no longer detectable at 12 months. However, frequently, some cases remain seropositive for several years after the treatment [194], particularly when starting with very high antibody titers [171].

#### Anaplasma phagocytophilum

Information regarding the persistence of *A. phagocytophilum* antibody titers is limited. However, some studies have described persistence of seropositivity for up to one year after the treatment [200].

#### Anaplasma platys

Information regarding antibody titers in dogs naturally infected with *A. platys* is scarce. A steady decline of antibody titers after therapy has been described in dogs experimentally infected with *A. platys* [90].

### Why is PCR useful after treatment?

PCR testing, when conducted several weeks after termination of doxycycline treatment, allows clinicians to be more confident that the treatment has been effective and that the dog did not enter the subclinical stage [6,142,146,186,201]. Yet, it must be recognized that a negative PCR result does not guarantee that the animal is “free of infection” (see above).

There are two goals for performing PCR some weeks after the completion of treatment. One is to minimize the probability of obtaining false-negative results, due to antibiotic effects on bacteremia. The second is to minimize the chance of detecting DNA of dead pathogens that continue to circulate during the treatment period [6,132,142,174,175].

In conclusion, PCR is useful for monitoring dogs treated for these diseases, because it can detect the presence of pathogen DNA, regardless of the serologic antibody titers. However, although this technique is more sensitive than serology in confirming an infection, its effectiveness may be limited for detecting pathogens in subclinically infected dogs, because the organisms may circulate intermittently in peripheral blood. Therefore, a PCR analysis should not be considered a definitive method for conclusively excluding subclinical infection in clinically normal dogs that remain seropositive after treatment [6,142,145,146].

### Can canine ehrlichiosis and/or anaplasmosis be cured?

In general, most acutely ill dogs infected with *Ehrlichia* or *Anaplasma* species heal after a specific, effective treatment of appropriate length. This finding has been corroborated with multiple clinical and experimental trials [166,168,170]. However, a recent work conducted with dogs experimentally infected with *E. canis* suggested that some dogs may remain persistently infected and may not eliminate the infection even after acceptable treatment [125].

### Can dogs be re-infected by *Ehrlichia* and/or *Anaplasma* spp.?

#### Ehrlichia canis

Re-infections of canine ehrlichiosis are possible, because no persistent or effective immunity develops to defend against re-infection with these pathogens [120]. In most cases, after treatment, the dogs return to the habitat they lived in prior to the infection; therefore, their exposure to infected ticks remains feasible. When no proper tick-control measures are employed, and the dogs are re-exposed, they can easily become re-infected. Moreover, mainly in cases of canine ehrlichiosis, it is difficult to distinguish a re-infection from recurrence of an established persistent subclinical condition [177].

#### Anaplasma phagocytophilum

It is not known whether a natural infection can provide long term protection against the development of clinical anaplasmosis. Re-infection following the therapeutic elimination of the organism has not been reported in dogs; however, in human medicine, one re-infection case was documented [202]. Conversely, horses have been shown to resist re-infection after recovery from an initial infection with *A. phagocytophilum* [200].

#### Anaplasma platys

The possibility of *A. platys* re-infections in dogs living in endemic areas and exposed to ticks has not been fully evaluated, but cannot be ruled out.

### What prophylactic measures can be implemented to prevent infection by *Ehrlichia* and/or *Anaplasma* spp. in dogs?

The prevention of ehrlichiosis and/or anaplasmosis infections in dogs must be focused on tick control. Ticks of the *R. sanguineus* complex are mainly found indoors, but other populations of the tick may be common in gardens, pastures, and fields in the Mediterranean basin. Therefore, dogs may become infected with the most common tick present in infested private gardens or kennels, but also, whenever they are engaged in an activity that involves contact with natural green areas. Nevertheless, to prevent transmission, actions must focus mainly on:

- preventing dogs from becoming infested in the field, which is the source of peridomestic parasitism. This infestation can introduce ticks to indoor habitats (kennels, etc.), which will result in a large population, due to their high reproductive capacity.
- preventing dogs from becoming infested with ticks, even when they live in a peridomestic environment with abundant ticks. This objective is more difficult to achieve than the former, due to the high parasitism pressure that can arise from those populations of ticks [203].

To appropriately control tick infestations in dogs requires knowledge of tick seasonality. This seasonality is usually strict under natural conditions, and each stage of the life cycle of the tick follows the previous stage with regularity. However, these patterns can be lost in areas with large infestations in a peridomestic environment. Under those conditions, all stages can be active simultaneously, due to the presence of several infestations in the kennel.

Outdoor populations of *R. sanguineus* can be active and, therefore infest dogs, when the average temperature is 10-12°C. Below this temperature, it is very rare that dogs acquire infestations from outdoor populations of that tick; consequently, during winter, it may be appropriate to reduce the treatment pressure against ticks. However, it must be noted that indoor populations of the ticks may be active year round, which adds extra risk for dogs living in kennels or dogs that spend time in dog day care centers. In Europe, tick control should be continuous between spring and autumn in areas where ticks are present. This pattern may vary according to the local weather and the historic weather particularities. Clearly, measures against *R. sanguineus* must follow extended schedules in the Mediterranean basin, because winters are shorter, and therefore, the periods of *R. sanguineus* activity are prolonged.

In the case of *I. ricinus*, the activation temperature for the ticks may be around 6°C. Therefore, adequate measures of control should be strictly followed to limit contact of dogs with ticks in infested areas [72].

Various antiparasitic treatments are registered in Europe that have activity against ticks and can be prescribed for dogs [204]. The specific product can be selected according to the preferences of use (collar, pour-on, or spot on) and the therapeutic needs for each case. Some compounds, such as the pyrethroids or some preparations of diazinon, are registered as repellents.

Apart from appropriate epidemiological measures, the best prevention against dog tick infestation is based on the use of ectoparasiticides. In general, these are active molecules that act against ticks, and they must also provide some degree of appropriate control within a short period of time [205]. Rapid action could contribute to the elimination of attached ticks before they are able to transmit pathogens. To optimize efficacy, preventive chemicals must be applied at the recommended time intervals, based on the length of efficacy claimed by the manufacturer. Although knowledge may be lacking for some diseases, most tick-borne pathogens require 4 to 48 h to complete their development in the salivary gland and enter the bloodstream [100]. However, a recent report showed that transmission of *E. canis* can occur within shorter periods of time (e.g., 3 h) [71].

### Are there vaccines available to prevent ehrlichiosis and/or anaplasmosis in dogs?

Currently, no commercial vaccines are available to protect against infections with *E. canis*, *A. phagocytophilum*, or *A. platys*. A recent study showed that an attenuated *E. canis* strain may serve as an effective future vaccine for CME [206].

## Conclusions

Information on canine ehrlichiosis and anaplasmosis in Europe has significantly increased in the last few years. This guideline aimed to answer common questions of interest on the etiology, transmission, epidemiology, clinical signs, laboratory findings, diagnosis, therapy, and prophylaxis of infections caused by *Ehrlichia* and *Anaplasma* spp. This guideline contributes to the understanding of the current status of these diseases on the European continent. However, these pathogens are spreading to new niches, and reports have described infections in many countries where they were not detected previously.

## Abbreviations

ACVIM: American College of Veterinary Internal Medicine
CME: Canine monocytic ehrlichiosis
ELISA: Enzyme-linked immunosorbent assay
ENTRA group: Abbreviation in Spanish for “*Grupo de Estudio de Enfermedades Transmitidas por Artrópodos*” – Arthropod-Borne Disease Experts Group
ESCCAP: European Scientific Counsel of Companion Animal Parasites
IFAT: Immunofluorescent antibody test
PCR: Polymerase chain reaction
PCV: Packed cell volume
PLT: Platelet concentration
APTT: Prolonged activated partial thromboplastin time
q12h: Once every 12 hours
WBC: White Blood Cells
